# ‘Team Speech Sounds’—How Speech and Language Therapists Work With Parents of Young Children With Speech Sound Disorder: A Focus Group Study

**DOI:** 10.1111/1460-6984.70224

**Published:** 2026-03-23

**Authors:** Katherine Pritchard, Vesna Stojanovik, Jill Titterington, Emma Pagnamenta

**Affiliations:** ^1^ School of Psychology and Clinical Language Sciences University of Reading Reading UK; ^2^ The Speech Doctor Holywood Northern Ireland UK

**Keywords:** behaviour change, home‐practice, parents, speech sound disorder

## Abstract

**Background:**

Speech sound disorder (SSD) is common amongst children on speech and language therapy caseloads, with most of these children being between 2 and 6 years old. SSD that persists into a child's school years can have a lasting impact on literacy development, socio‐emotional outcomes and well‐being. Effective intervention in the preschool years is therefore vital. Evidence shows speech and language therapists (SLT) believe working with parents is essential for a child's progress, and positive relationships between SLTs and parents facilitate engagement with home practice. However, little is known about what practising SLTs do to support parents to deliver effective home practice or how they form positive relationships with parents.

**Aims:**

In this study, we explored SLTs perceptions and experiences of their current practices when working with children up to 5 years and 11 months with SSD and their parents. Our focus was on the techniques and strategies SLTs use to support parents to implement effective intervention at home with their child, how they develop effective working relationships with parents and the barriers and facilitators to supporting parents effectively.

**Methods and Procedures:**

We used a qualitative, focus group methodology. Fifteen SLTs, recruited via social media and professional networks, participated across four 2‐h online groups. Participants had a range of years of clinical experience, worked across public and independent sectors and came from different geographical locations. Groups were recorded, transcribed verbatim and analysed using Reflective Thematic Analysis.

**Outcomes and Results:**

We constructed four main themes: (1) Individualisation, flexibility and sufficient time allow for an accessible service. (2) SLTs’ individual circumstances and attitudes influence how they support parents. (3) SLTs work to ensure the fidelity of home practice. (4) Nurturing relationships is fundamental.

**Conclusions and Implications:**

We demonstrate that what SLTs do to support parents aligns with the COM‐B model of behaviour change: SLTs develop their own and parents’ capability; they individualise provision to allow parents the opportunity to access it and build relationships to support motivation. This works towards the desired behaviour—engagement with and fidelity of home practice. Our study builds on emerging research into the use of behaviour change theory, highlighting building relationships as fundamental to SLTs and parents. SLTs need to reflect on the impact of their own personal circumstances. Support for parents needs to be individualised to create opportunities, while ensuring adherence to the evidence so that home practice is delivered with fidelity.

**WHAT THIS PAPER ADDS:**

*What is already known on the subject*

Speech sound disorder (SSD) is common in Speech and Language Therapist's (SLT) paediatric caseloads. SSD that persists into school years can have a long‐term impact. SLTs believe that working with parents to support home practice is important, but little is known about what practising SLTs do to support parents to deliver effective home practice, how they form positive relationships with parents or how they perceive working with parents.

*What this paper adds to existing knowledge*

Our study explored how SLTs work with parents to ensure home practice is delivered with fidelity and what they do to build effective relationships. Our findings suggest that SLTs use strategies which align with behaviour change theory, by building on parents’ and their own capability and, by adapting to individual families to maximise opportunities for parents to become effective implementors. Importantly, building relationships with parents not only supports the motivation of parents but also of SLTs.

*What are the potential or actual clinical implications of this work?*

Our results suggest that relationships between SLTs and parents underpin effective home practice. Developing these relationships takes time and skill. Building relationships with parents and helping them to understand what they need to do and why is likely to have a positive impact on the parents’ ability to implement home practice. These relationships may allow SLTs to skill up parents more effectively and support the individualisation of the intervention approach, which is important to both parents and SLTs. SLTs should be cautious however to ensure that the boundary between adherence to the evidence base and effective individualisation to optimise outcomes for the child is maintained.

## 1 Background

Children with speech sounds disorder (SSD) can have difficulties with any combination of ‘perception, articulation/motor production, and/or phonological representation of speech segments (consonants and vowels), phonotactics (syllable and word shapes), and prosody (lexical and grammatical tones, rhythm, stress, and intonation) that may impact speech intelligibility and acceptability’ (McLeod et al. 2013, 1). SSDs are common in the paediatric population, with reported prevalence being anywhere from 2.3% to 24.6% (Beitchman et al. 1986; Eadie et al. 2015; Jessup et al. 2008; Law et al. 2000; Shriberg et al. 1999). The variation being due to differences in how SSD has been defined and what age range has been considered. Broomfield and Dodd (2004) found that 85% of referrals for children with SSD to a community paediatric service in the United Kingdom were for children under 6 years. Children with SSD past age 8 are more likely to report self‐harm with suicidal intent (McAllister et al. 2023), have poorer academic outcomes (Wren et al. 2021) and difficulties with peer relationships (Wren et al. 2023), all making effective intervention in the preschool years vital.

Existing evidence suggests that direct intervention with a Speech and Language Therapist (SLT) is necessary for children with a range of SSD diagnoses (McCabe et al. 2020; RCSLT 2024; Sugden et al. 2018). Intervention is more effective when delivered with higher frequency (e.g., Allen 2013; Thomas et al. 2023). For example, Allen (2013) found that children with Consistent Phonological Disorder, who were provided with Multiple Opposition intervention three times a week, made significantly greater gains than those who were offered it once a week. Thomas et al. (2023) concluded that weekly sessions for Rapid Syllable Transition, an intervention for children with Childhood Apraxia of Speech, were not recommended, compared with previous studies showing its effectiveness when delivered more frequently than this (Thomas et al. 2014). Despite this research evidence, there is a gap between the intervention intensity provided in practice and what is reported in the research (e.g., Hegarty et al. 2018; Oliveira et al. 2015; Sugden et al. 2018).

Therapy can be effective when direct intervention from an SLT is supported by a trained adult (i.e. a parent) through home practice. For example, Sugden et al. (2020) in their multiple opposition intervention study asked parents to complete home practice twice per week, following explicit training. Home practice took the form of phonological awareness tasks, drill‐based speech activities and shared book reading. Parents were provided with comprehensive training before the intervention, through a dedicated parent‐only training session, plus training within the sessions via observation, practical application of the intervention with their child, feedback from the SLT and chances for self‐reflection. Parents’ observation skills were trained in the sessions using a structured observation sheet of the SLT working with their child. These sheets were also used to guide the SLTs’ feedback to the parents. The parent training session supported the parents to understand their child's diagnosis, the intervention approach and the expectations for home practice. The parents were also provided with written information and were supported to perceive speech errors via targeted listening activities. Parents were asked to record when they completed home practice and what activities they did, with some reflection on how it had gone. Scherer et al. (2008) asked parents to complete 10–20 min of focused stimulation every day. Training for this involved supporting the parents’ understanding of the intervention verbally (supported by written information), chances for role‐play with scripts, observation of the SLT and a chance to try with their child, with SLT feedback provided. Between two and four 45‐min training sessions were provided to ensure the parents were 80% accurate in their delivery of the intervention before requiring them to complete home practice. Both studies highlight the importance of explicit training of parents within direct sessions with an SLT prior to home practice. Many other studies that include home practice in the literature are lacking in methodological detail (Sugden et al. 2016). This means that it is often unclear how SLTs have ensured the fidelity of the home practice or what the potential facilitators and barriers are.

SLTs believe working with parents as implementors of intervention is vital for children with SSD to make progress (Sugden et al. 2018; Watts Pappas et al. 2008). Professional best practice guidelines recommend that SLTs work with parents (RCSLT 2018), and it is recognised that getting the relationship between parents and SLTs right in the early stages of therapy is crucial (Melvin et al. 2020). Relationships are also important to parents (Watts Pappas et al. 2016). However, currently SLTs have little understanding about the best ways to effectively work with parents (Sugden et al. 2018). Working with parents is thought to be central to success in therapy for children with SSD (Furlong et al. 2018) thus making this an important area for further investigation.

Existent literature has mostly explored how SLTs work with parents of children with SSD, using survey‐based research (Hegarty et al. 2018; Sugden et al. 2018; Tambyraja 2020; Watts Pappas et al. 2008). These surveys do not provide specific details on how or why therapists work in the way they do. While some studies discussed the perceived barriers to supporting parents, such as parental capability, they have not explored the reasons or how SLTs try to overcome these barriers to support parents to deliver home practice (Sugden et al. 2018; Watts Pappas et al. 2008).

There is a link between parental beliefs in the importance of a recommendation and their adherence to the recommendation at home (Williams et al. 2024). This further emphasises the importance of SLTs supporting parents to understand why they are being asked to complete home practice. However, there is currently limited knowledge about how SLTs perceive working with parents, how they support them to deliver home practice effectively and how they develop relationships to support this home practice, for children with SSD (Pritchard et al. 2024).

## 2 Aim

In this study, we explored SLTs perceptions and experiences of their reported current practices when working with children up to 5 years and 11 months with SSD and their parents. Our focus was on the techniques and strategies SLTs use to support parents to implement effective intervention at home with their child, how they develop effective working relationships with parents and the barriers and facilitators to supporting parents effectively.

## 3 Methodology

We took a subtle realism approach (Hammersley 1992), starting from the underlying reality of SLTs and parents working together, looking to represent this reality through the views of SLTs, whilst also recognising that we as researchers and K.P., E.P., J.T. as SLTs ourselves influenced the research. We used a qualitative, single‐category design, focus group methodology (Howitt and Cramer 2020). Ethical approval was obtained from the University of Reading Research Ethics Committee [2023‐087‐EP]. Focus groups were chosen to allow for a dynamic discussion, to exploit the interactive potential of a group situation and to allow us to gather a rich and detailed data sample (Howitt and Cramer 2020). We report the data using the Reflexive Thematic Analysis Reporting Guidelines (Braun and Clarke 2024) (in Supporting Information).

## 4 Participants

Participants were all qualified SLTs, who currently work with children with SSD, younger than 6 years of age, and their parents, or have done so in the last 12 months. We conducted the groups online via Microsoft Teams to increase the reach of the study and ease of participation (Lobe et al. 2022).

## 5 Sampling and Recruitment

Participants were recruited from across the United Kingdom and Channel Islands^1^ via email, professional networks, advertising on social media and snowballing. We used this method to ensure clinicians from different sectors were recruited (public, independent, and higher education), clinicians with varying years of experience, and those from a wide geographical area. This allowed purposeful sampling of the groups to ensure a wide range of views were heard (Tritter and Landstad 2020).

Our sample size was guided by recommendations to run 3–4 groups (∼4 participants per group) within a single category study design (Howitt and Cramer 2020). This was deemed to provide the information power required to ensure the data was robust and trustworthy (Malterud et al. 2016). We took an iterative approach to the data to determine its richness and discussed whether the data was sufficient to meet our research aims as we progressed, as recommended by Braun and Clarke (2021).

## 6 Data Generation

K.P. facilitated four, 2‐h focus groups with E.P. present as a silent observer, to make field notes and support with the technology, as recommended (Lobe et al. 2022).

The focus groups addressed three research questions, developed from the study aim and were guided by a semi‐structured topic guide.

1. What are the perceptions and experiences of SLTs about the way they currently work with parents, within therapy sessions, to ensure that the approach and intensity of intervention are delivered with fidelity in the home environment?
2. What do SLTs think works well to build relationships with parents to support them with home practice for SSD therapy?
3. What do SLTs think are the barriers and facilitators to supporting parents effectively?

See Supporting Information for the full topic guide. K.P. designed the guide, following a review of the literature (Pritchard et al. 2024), in consultation with the research team, who drew on their clinical skills and expertise.

Groups were audio/visual recorded via MS Teams. Automatic transcripts were checked by K.P. for accuracy and transcribed verbatim with identifying details removed.

## 7 First Author's Reflexivity

Throughout the study, we took a reflexive approach with K.P. keeping a reflexive diary and regular discussions with the whole team. See Appendix 1 for a detailed reflexivity and positionality statement.

## 8 Analysis

As a qualitative study, focused on human experiences, our initial data analysis was predominantly inductive to allow for new and novel concepts to be generated (Bradbury‐Jones et al. 2017). Later stages of our analysis took a mixed inductive/deductive approach, comparing the analysis to the existing literature, and ensuring the research aim was addressed by the themes. We used six stages of reflective thematic analysis (RTA) to guide the process. Our approach was not linear but went back and forth fluidly between the data, codes and themes to ensure the outcome of the analysis made sense in relation to the data (Braun and Clarke 2021). We developed semantic and latent codes with no prioritisation of one over the other (see Appendix 2 for examples). All analysis was completed by K.P. and discussed with the rest of the team to refine thinking and sense check the narrative of the analysis. We chose RTA as K.P., like the participants, is a clinically active SLT, with a specialist interest in SSD. It was therefore important to take a reflexive approach to analysis to ensure we were mindful of how our experiences shaped the data generation and analysis. Analysis continued until we were happy that the theme names reflected the true nature of the shared meaning (see Appendix 3 for an example of the evolution of Theme 2)

Towards the later stages of the analysis, we used the COM‐B behaviour change model (Michie et al. 2011) as a lens with which to frame our results. This model examines the capabilities (C), opportunities (O), motivations (M) that influence behaviour (B) change. ***Capability*** refers to an individual's capacity to engage in an activity; ***opportunity*** to factors that are outside of an individual that make a behaviour possible; and ***motivation*** is either conscious or sub‐conscious factors that can impact behaviour. The interest in using such frameworks to examine SLT interventions has been growing in recent years (e.g., Baker et al. 2024; Barnett et al. 2023). Within this research, we were interested in applying the model both to the SLTs’ own behaviours and to how SLT's develop parents’ behaviour to support home practice. This model allowed us to look at the parental behaviours targeted by the SLT participants (B), how the SLTs described their own capabilities and how they used their skills and knowledge to develop the parents’ capability (C), the opportunities SLT's had for supporting parents and how they provided opportunities for parents to support their children (O) and theirs and the parents motivation for working together (M) (Michie et al. 2011).

(See Supporting Information for a more detailed description of our analysis methodology, including reflections).

## 9 Results

Fifteen participants who worked in a variety of sectors, geographical locations and with a range of years of experience as SLTs participated in the focus groups. For full participant characteristics, see Table 1.

**TABLE 1:** Participant characteristics (n.b.—there was incomplete data from one participant).

Characteristics	No. of participants total *n* = 15
Years of experience:	
Less than 5	1/15
6–10 years	4/15
11–15 years	4/15
16–20 years	1/15
More than 20	5/15
Sector:	
NHS	11/15
Independent	3/15
University	3/15
Education	1/15
Public health (not NHS)	1/15
More than one sector	3/15
Self‐reported expert in SSD	11/15

Location	
East of England	2/15
London and South East England	9/15
West Midlands	1/15
Channel Islands	1/15
North East England	1/15
Other	1/15

Sex	
Female	14/14
Male	0/14

Ethnicity (self‐reported based on Office for National Statistics classifications)	
White British	11/14
Other white background	2/14
Mixed/multiple ethnic groups	1/14
Parent	9/14
Multilingual	4/14
Known to KP/EP prior to groups^5^	4/15

**FIGURE 1:**
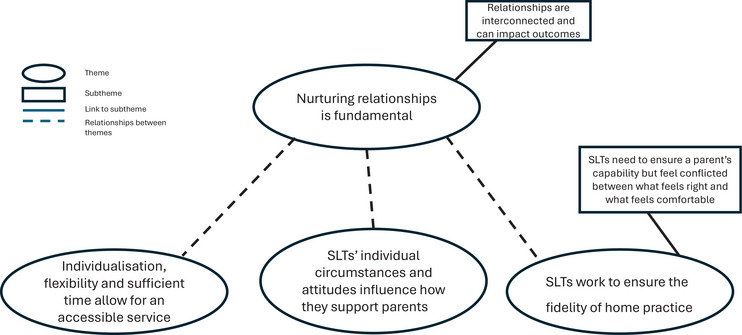
Diagram of themes (amended).

**FIGURE 2:**
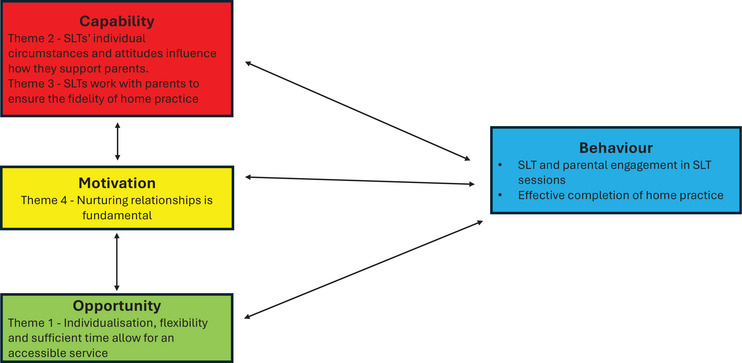
Themes within the COM‐B model (amended).

We explored practices and perspectives of SLTs working with parents of children under 6 with SSD, focusing on how they work with parents within intervention sessions to support effective home practice; how they build working relationships with parents, and the barriers and facilitators to supporting parents well. We constructed four themes and two sub‐themes (Figure 1). We examined our four themes within the COM‐B model (Michie et al. 2011) to explain how SLTs target the desired behaviours with parents. We found that all areas of the model were relevant to our findings and interlinked with each other, with increased ***capability*** and ***opportunity*** allowing for increased ***motivation*** and vice versa (see Figure 2).

### 9.1 Theme 1—Individualisation, Flexibility and Sufficient Time Allow for an Accessible Service

Within this theme, we considered the ***opportunities*** available to SLTs and how they support ***opportunities*** for parents to impact and influence the desired behaviours. This theme also examines SLT's ***capabilities*** to individualise their approach and to integrate the research evidence within this.

To ensure diverse families have an equal opportunity to benefit from their speech and language therapy provision, participants felt that providing a flexible and individualised service was essential. Previous studies have found that individualisation is vital to success for families (Furlong et al. 2018; Klatte, Bloemen, et al. 2024; Klatte, Ketelaar, et al. 2024; Sugden et al. 2019). The decisions SLTs make about how to individualise and thus how they make the service accessible to the family are multi‐faceted (e.g. Furlong et al. 2018; Sugden et al. 2019). Our participants often supported increased ***opportunities*** for parents by balancing the research evidence with the individual's needs.

‘Following sort of like the evidence‐based sort of published approaches, but then also sometimes it's devising sort of a techniques that are just very specific for individual children that there wouldn't be anything written up in the research’. P5 (university, 20+)^2^

This is in line with previous studies that have found SLTs, deliver intervention that mix aspects from several different therapy approaches (Hegarty et al. 2018, 2021; Joffe and Pring 2008). This approach is described as ‘hybrid’ (Hegarty et al. 2018, 1002) and some potential rationales that are given for this are service restrictions and SLTs’ lack of knowledge. However, as suggested by the above extract, our participants use their ***capabilities*** informed by all aspects of evidence‐based practice to consider the research evidence, their knowledge of the client and their own clinical experience/expertise (Sackett 1997) to think carefully about what they do, ensuring that there are the maximum ***opportunities*** for intervention and home practice by tailoring the approach to the individual. Conversely, it should be noted that these decisions are often made in the context of restricted services in which our participants perceived that adhering to the evidence base is not possible. As well as combining strategies and approaches, some of our participants spoke about reducing the frequency of sessions based on parental preferences, which has the potential to reduce the effectiveness of the intervention by decreasing the intervention intensity. Combining methods in the context of a restricted service or adapting intervention intensity based on parental preference raises potential concerns about the efficacy of clinical practice. Could individualising an approach, especially when considering frequency of sessions, veer too far from the evidence base and prove ineffective? And what happens to an SLTs ability to individualise intervention when time with a family is restricted by the services they work in? These factors are especially pertinent when considering the amount of time needed to develop relationships and gather the information required for effective individualisation.

Klatte, et al. (2024) systematic review of collaboration between SLTs and parents found that treating every family as unique by taking time to get to know the parents’ background and evolving needs contributed to successful collaboration. This is reflected in our participants who felt that having time and thus the ***opportunity*** for flexibility in the way in which they work, allowed them to provide a service which was accessible to a range of families. There was a sense from participants that they had to constantly reflect and work to ensure they provided an accessible service. To ensure ***opportunities*** for this reflection, they needed time within the service which was not always given to them. Without time SLTs felt restricted in terms of how well they could support parents to engage in the intervention process and take on the role of implementor of intervention at home and this impacted their perceived effectiveness of what they were providing.

‘If after every session I had the time to properly sit down and think, what could we do better? How could we get parents more engaged? […]^3^ you don't really have that time to kind of problem solve where to go next with a lot of the children’. (P14 – NHS^4^ 5–10)

This comment reflects how hard a lack of time can be for SLTs, and this can lead to a sense that they are not doing their job well. It restricts the ***opportunities*** that the SLT can create for parents and thus runs the risk of being ***demotivating*** for both parents and SLTs.

However, not all participants felt restricted and were able to reflect on the benefit of having the time and flexibility within their service:

‘I'm really lucky in my service we are able to offer children what they need’. (P2 NHS 10–15)

P2's comment, when compared to P14's, demonstrates that within the NHS there can be disparity, and how the service is run can impact the time an SLT has and the service they provide. The word ‘lucky’ from P2 indicates that they are aware that this level of service, where they have enough time to offer children what they need, is not the norm in the United Kingdom, and they are in a special position. This sense of privilege and even guilt was consistent across the participants, who indicated that they had what they needed to be flexible.

These findings demonstrate how a lack of flexibility and time within a service reduces the ***opportunities*** for SLTs to individualise their approach, thus also restricting the ***opportunities*** available to the parent.

### 9.2 Theme 2—SLTs’ Individual Circumstances and Attitudes Influence How They Support Parents

In this theme, we look at an SLTs own ***capability*** and how this impacts how able they are to develop a parent's ***capability***. All SLTs come to their practice with differing knowledge, skills, perceptions and experiences, and our study found that these can all have an impact on how they support parents.

Most participants shared the view that the more experienced they got, the better they were at supporting parents to develop their ***capability*** within the therapy sessions. They reflected on how much there was to think about when they were newly qualified and how little capacity they had, within a session, to consider parents when they did not feel confident with what they were doing themselves. P2 reflected that:

‘I think that [experience] makes a huge difference and so now, as a more experienced therapist, I would go into all of my sessions with parents, thinking in my head, how can I coach them?’ P2 (NHS 10–15)

This comment reflects how increased ***capability*** developed through clinical experience strengthens the ability to work collaboratively with parents and can allow SLTs to be mindful of the parents’ experience of the service, whilst still delivering the intervention required.

When asked if the university prepared them to work well with parents, P5 commented:

‘The way I was trained as a student was always to work through the parents. I don't know anything different’. (university, 20+)

However, for the most part, SLTs felt that their university education did not prepare them for working with parents and that they were dependent on the ***opportunities*** provided to them via their clinical placement experiences to develop their ***capability*** with parents. They reflected on the way they had been able to engage in continuing personal and professional development (CPPD). This reflection included how they needed to draw on other ***opportunities*,** such as being or becoming a parent or a previous job role, as well as transferring skills from their reading, post‐graduate training and experience from working with other client groups. This is demonstrated by the extract from P7, who is reflecting on applying skills learnt from parent‐child interaction support (PCI) to working with SSD:

‘What I do has come from the literature that I've read around that [PCI] and actually like in PCI, how the coaching element is so important and that's why I kind of started to then think actually that must be the case of the speech sound ones’. (NHS/private 5–10)

P7 reflects here on how the evidence base from a different client group may also apply to their work with parents of children with SSD, demonstrating how SLTs use their skills in reflective practice to develop their ***capability***.

As well as the impact that experience and knowledge have on an SLT's ***capability***, our study found that an SLT's values and beliefs have the potential to make a difference to the way in which they work with parents. This is reflected in Klatte et al. (2024) who found that nearly all the papers they reviewed considered the SLT's mindset as important to successful collaboration.

Whilst, as reported in the existing literature (Sugden et al. 2018; Watts Pappas et al. 2008) our participants agreed that *‘it's vital that we work with parents’ P9* (NHS 20+), participants did not agree on a variety of things, including what their role was and the role of home practice. Such differences in attitudes are demonstrated here by comments from two participants about the role of the SLT and the parent within the therapeutic process. P5 felt very strongly that they were:

‘An equal participant with the with the parents’ and that ‘ultimately it always comes down to us, as the therapist to find the way forward’, (university 20+)

This indicates the value they place on working with parents and how parents can support the child. It also reflects on a sense of responsibility they feel towards providing ***opportunities*** to the parent to ensure the success of the intervention and the belief that their role includes problem solving and decision making when things are not working. Conversely P13 commented that:

‘It's the parents’ responsibility at the end of the day, if they are concerned enough and want the help, they can access it’. (NHS 5–10)

This participant felt that it was the parent's responsibility to access the ***opportunities*** provided, seeing it as the parent's role to find the way forward and thus potentially influencing the way in which they approach working with parents. Putting the onus on parents to navigate their way through the service has the potential to make intervention hard to access for some families due to a lack of ***opportunity*** being created by the SLT. These conflicting opinions highlight the role that the SLT can have in facilitating or blocking access to and engagement with a service.

Participants also disagreed on the role that home practice plays in a child's SLT care. It can be seen either to ‘top‐up’ sessions that they are unable to provide in their model of service or as a way to generalise the child's skills into their everyday life. Some felt that supporting parents to become implementors of intervention was the only way forward due to a lack of ***opportunity*** and this made them feel helpless, as can be seen by P7's comment:

‘I think we we haven't got a choice but to ask parents to support us, or education settings as well, in terms of increasing that intervention intensity’. (university/private 5–10)

The sense that this is the only way and the implication that this is not how they would like to work could impact how they approach the sessions. On the other hand, P8 was clear that home practice was essential no matter what and commented:

‘It's not about increasing the intensity; it's about making those changes because you can't make those changes. I can't sit on a child's shoulder and correct them continuously. It's got to be a joint teamwork around the child’. (NHS 20+)

The SLT's reference to ‘continuously’ correcting the child implies that the parent is essential to the feedback given to the child outside of the intervention sessions to support the generalisation of skills. Seeing home practice in this way, as an important part of a child's intervention may contribute to an SLT's ***motivation*** to work with parents effectively. This can then also have an impact on the parent's engagement as found in past research where the SLT's attitude in relation to the parent's skills and a lack of respect for these can impact how a parent approaches the intervention (Watts Pappas et al. 2016). An SLT's attitude towards working with parents therefore has the potential to either be facilitative or a barrier to providing ***opportunities*** to parents and thus developing their ***capability***.

### 9.3 Theme 3—SLTs Work With Parents to Ensure the Fidelity of Home Practice

For successful home practice SLTs develop parents’ ***capability*** to ensure they can deliver home practice that is as faithful to the evidence as possible. Our study found that expectations of both the parents and the SLT matter to the success of home practice. Some participants felt that parents often come to the therapeutic process with different expectations of it to them. They felt parents saw the SLT role as ‘fixing’ and that it was therefore important to discuss and set expectations.

‘To get the parents on board straight away to see that it's not me as the speech therapist, (P10 nodding) it's going to fix the child that it's a, you know, it's a shared responsibility’. P9 (NHS 20+)

This comment reflects thoughts from across the groups that these expectations need to be set from the beginning to ensure that everyone is clear of their role to support the child. The importance placed, by the SLTs, on parents understanding their role and expectations indicates that our participants felt that this understanding would support parental ***motivation*** to participate in the intervention, in particular with the home practice.

Having shared expectations has previously been found to be important when working with parents, both from the SLT (Furlong et al. 2018) and the parental perspective (Sugden et al. 2019). However, these expectations do not always translate and parents can be left feeling like the SLT has expectations that they are not aware of (Sugden et al. 2019). Our participants discussed overcoming this by ensuring they embed ***opportunities*** in their sessions to discuss clear expectations and to support parents’ ***capability*** and ***motivation*** by understanding why they need to do home practice and the amount needed for their child to progress.

‘If you have medicine, you have to take it X number of times a day or a week and it's kind of similar and I talk to them [parents] about, you know, what is, has been shown to be effective and how we can work together’. P7 (NHS/private, 5–10)

Using analogies, such as this, and making explicit references to the literature were techniques mentioned to support a parent to understand why they are doing something. Previous literature suggests that parents’ expectations and views of their role in intervention can change over time (Davies et al. 2017). Parents understanding the rationale for an intervention is key to successful collaboration between SLT and parent (Klatte et al. 2024) and as seen within our study can support parental ***motivation***. Parents understanding why also supports parents’ ***capability*** to deliver home practice with fidelity (Pritchard et al. 2024).

#### 9.3.1 Subtheme 1—SLTs Need to Ensure a Parent's Capability but Feel Conflicted Between What Feels Right and What Feels Comfortable

To ensure the fidelity of home practice SLTs need to ensure not only that parents are completing the home practice but also that they are doing so correctly. It is the SLT's role to provide ***opportunities*** within the session to discuss home practice with parents, however Tambyraja (2020) found follow up of home practice was missing in SLTs sessions. Contrary to Tambyraja's finding our participants reflected on a variety of ways in which they provided this ***opportunity*** through monitoring what parents were doing and how they were doing it. These included use of tick sheets, sticker charts, video feedback and discussion. Like in previous studies, for example, Furlong et al. (2018) and Sugden et al. (2020) monitoring the accuracy and amount of home practice was an important consideration for our participants but they acknowledged that this could feel uncomfortable and could therefore impact their ***motivation*** to do so.

Observation of the parent working with their child came up across the groups as one technique to assess and monitor the parent's ***capability***. Following an observation, SLTs often provide feedback to shape what the parent is doing. There were mixed feelings about this across the groups:

‘I'll watch them do the activity that I want them to try and then kind of say well, I think you did really well at that or maybe we could just tweak this, that was brilliant, tweak this bit so that then they feel confident leaving that they can do what the therapist does’. (NHS 20+)

Here P8 uses observation and feedback to not only monitor the accuracy of what the parent is doing but also to reassure the parent they are doing the right thing. The outcome of this is parental confidence. Instilling this confidence provides a ***motivating*** factor for the parent to complete home practice as an effective implementor.

However, not everyone agreed that observation works to boost parents’ confidence. As seen here from P11:

‘Some parents don't want to get it wrong or they, you know, they're not comfortable in that sort of environment to to be coached’. (NHS 20+)

They are reflecting that whilst observation works for some, there is a need to adapt the way they monitor and support individual parents to develop their ***capability*** to become skilled implementors of intervention. Here they reflect that being watched by an SLT could be a negative or ***demotivating*** factor for some parents, rather than instilling confidence as suggested by P8.

For others the discomfort was experienced by the SLT, rather than the parent. Discussion led P6 to reflect on their feelings about observing parents:

‘I always pull back from it [observing parents] because I have this feeling of, this is going to make the parents feel like they are being examined. […] it's just I need to break that. I think it is exactly what we need to do, but I I feel like I'm putting them [parents] on um, yeah, trial […]. Yeah, it feels uncomfortable to me. Probably wouldn't feel uncomfortable to them, but it's interesting to hear that you do that and it seems fine’. (private 10–15)

There is a sense of conflict here between the ‘right’ thing to do to ensure fidelity and the SLT's sense of comfort. This comment also suggests the possibility that the discussion in the focus group may have supported them to rethink this view.

Another source of potential discomfort for the participants was around the use of videos. These can be used to enhance a parent's ***capability*** by providing feedback to the parent. Some participants saw the value in using video as discussed by P7:

‘I find so many of them [parents] forget, particularly when they're not ones where actually the child is just getting it and they can go off and do it […] I've had feedback that's really helpful that they've been to watch the videos back and know what they were trying to get’. (NHS/private 5–10)

They describe video as a useful tool which allows parents to refer to the session, thus increasing the accuracy of their practice. However, some participants were not comfortable with using video, worrying about parents having videos of them.

‘That's one thing I I have never done. I think I'm very concerned about where those videos could end up and how do you actually sort of manage that’. P5 (university 20+)

This demonstrates how SLTs must navigate their practice in the age of social media where parents can share videos widely. SLTs need to balance the tools they use and how they work with parents with issues such as ensuring appropriate consent, safe storage and compliance with General Data Protection Regulation (GDPR).

Our findings suggest that even when SLTs have the ***capability*** to support parents to develop their skills and knowledge they are not always ***motivated*** to do so due to feelings of discomfort or concerns about potential wider impacts.

### 9.4 Theme 4—Nurturing Relationships Is Fundamental

Relationships between all participants in the therapeutic process has been found to be important for children with SSD and their parents (Sugden et al. 2019; Watts Pappas et al. 2016). It has also been found as important for parental engagement within speech and language therapy (Melvin et al. 2020). This theme threads through all the other themes and is overarching. Without relationships it would not be possible to get to know the family in a way that allows ***opportunities*** for an individualised and thus accessible service; SLTs require the ***capability*** to build relationships developed from their experience, skills, knowledge and the right attitude; and this connection is needed to build trust which ***motivates*** and supports parents to deliver effective home practice (the desired ***behaviour***). Relationships in themselves can also be a ***motivating*** factor for parents, children and the SLT to drive the desired behaviour.

Some of our participants were enthusiastic about working with parents. The passion they have for their jobs came through when they were discussing how they built partnerships with the parents.

‘It's a beautiful thing to be able to give parents the magic moment when they do something and it works […]. And I think to be able, as human beings, to be able to give that […] they've [parents] often been through a lot and it's just as a human being, lovely to be able to empower to, to not to empower, but to give parents that opportunity to do that with their child and to see that happening is a lovely thing to, to be able to do’. (private 10–15)

P6 reflects on the enjoyment they get from supporting the parents and watching the skills progress and then the success that this brings. This makes their job enjoyable and gives a sense of partnership between them and the parents, working towards a common goal. There is a sense from this participant that the relationship with families is what ***motivates*** them to include parents in their sessions.

Other participants felt that getting parents on board as partners was not always easy and at times, not possible. They have experienced some parents not engaging with intervention and this is a barrier to building the partnership between SLT and parent.

‘They [families who don't engage in SLT] often get discharged and the ones you feel really guilty about discharging, (P13 and 15 nod)’. P14 (NHS 5–10)

We are unclear whether discharge in this discussion is triggered by the service's policies and procedures or whether P14 feels that this is the only option for families where the engagement is low. Discharging families based on engagement poses a potential risk to children with SSD whose parents cannot access a service or who need more support than is routinely given. It is possible that more flexibility in how the service is delivered, including time built in to support relationship development, could in turn support parental engagement for families where building relationships and adhering to expectations of home practice is harder. However, there is a sense of helplessness here and it appears that P14 does not want to discharge these families but is not sure what else to do when they are unable to build a partnership. They are resigned to this discharge, as are P13 and 15 who nod along in acknowledgement.

The environment and resources participants have available to them can also impact on their ability to build a partnership with the parent. Participants discussed working in rooms that felt medical, set up of the chairs and organisation of the resources used but P9 shared a story that illustrates this point clearly:

‘All we had in the clinic was some black and white pictures and some old toys and this Dad went, you know, he just sort of lent forward, he goes, seriously? For real? Is that as good as you've got? I feel like I'm in a time warp. This is like the 1950s. And he walked out’. (NHS 20+)

The lack of modern resources within the clinic in this example put the parent off straight away, the father was not ***motivated*** to stay and P9 was therefore unable to begin building the partnership. This demonstrates how SLTs need to consider not just their interpersonal skills when building partnerships but also the physical environment.

#### 9.4.1 Sub‐Theme 2—Relationships Are Interconnected and Can Impact Outcomes

Parents of children with SSD have identified the importance of all the relationships in the therapeutic process (Sugden et al. 2019; Watts Pappas et al. 2016). The SLTs in our study supported this view which is demonstrated by P6's response to ‘*What supports positive relationships between SLT and parent?*’

‘I have never met a parent yet who didn't respond positively to me finding positives in their child and particularly for the children where actually life is hard for them and lots of things are difficult. (P5 nods along) Finding things to comment on that are positive can actually be really nice for a parent and and there's always something isn't there? […] It's never too hard to find, even with the child's bouncing around the room, and you know, not wanting to do what you want them to do. There's always something that you can find that's a positive, and I think for me that's one of the key things. Is just understanding what's brilliant about their child and verbalising that. So that's often where I when I start, I think’ (private 5–10)

By building a positive relationship with the child and demonstrating this to a parent P6 believes that they are also working to build their relationship with the parent. Their comment indicates that for some children, SLTs may have to work harder to find these positives, but they believe that it is possible in all cases. This is even more important for children who have the greatest level of support needs and P6 is ***motivated*** to find the positives for the benefit of the relationship. This supports the existing evidence suggesting the interconnectedness of parent‐SLT‐child relationships (Sugden et al. 2019; Watts Pappas et al. 2016).

Across the groups there was discussion about the importance of these relationships in relation to the outcomes for the children. P3 (NHS 5–10) referred to themself, the parent and the child as ‘*team speech sounds*’ indicating how integral each member is to the intervention. However not everyone agreed that relationships made a difference to the outcome of the intervention. For some, the relationships between the different parties were vital and could make the difference. P11 described a child who had been coming for intervention for several years and up until this point was making slow progress.

‘Then something changed at home and the dad started working with him and he made amazing progress because there was this special relationship between him and his dad, he suddenly had this time, how I interpreted it was, he suddenly had time with his dad and and he, it motivated him more because it was it was, you know, he had so much therapy, he often would be like, oh, can't do it, my mouth hurts, when he was with his mum and then with the dad it was just a different sort of relationship between them and I I was that very much kind of like, you know, you get on, you know, I was just a facilitator, which is the way it should be’. (NHS, 20+)

P11 attributes the success of the intervention to a change in the implementor of the home practice and the positive relationship they have with the child. This also demonstrates how a positive parent‐child relationship can impact on the SLT. P11's relationship with the child and parent changed as their role changed because of the relationship between father and son which they see as a change for the better. P11 also makes reference to how the relationship between father and son was a ***motivating*** factor for their home practice.

Conversely other participants felt relationships do not necessarily impact on the outcome of intervention and participants reflected that, in some circumstances, the relationship does not make a difference, especially when there is a lot happening for the family outside of the SSD, such as difficulty finding housing. In these circumstances participants felt that there was nothing they could do to improve the engagement in home practice and thus the outcomes. There was also concern amongst some people that a relationship between SLT and parent could become too close, and this could have a negative impact on outcomes.

‘I think sometimes they [parents] can get too comfortable with you and then they'll go the other way and go ‘no I haven't done anything this week’ and then you're like, come on. I thought we need to do something next week. No, still haven't done anything. So I think there's a bit of a fine line’ P7 (NHS/private 5–10)

P7 feels that whilst the relationship can be a facilitator it can also mean that parents do not feel ***motivated*** to complete the home practice due to not being accountable, feeling that it is acceptable to not complete it, thus having a negative impact on the outcomes.

## 10 Discussion

Our study set out to explore SLTs perceptions of and beliefs about their current practices with parents. Focusing on therapeutic relationships; the ways in which SLTs work with parents; how they perceive that these techniques and strategies support parents to deliver home practice effectively; and their beliefs around barriers and facilitators to supporting parents effectively.

We used the COM‐B model (Michie et al. 2011) to consider our results and what the driving factors of the SLTs’ practice may be in changing parents’ behaviour. Whilst our participants may not have been aware of this model and this was not explicitly discussed with them, the themes generated broadly fall under ***capability, opportunity*** and ***motivation***.

### 10.1 Capability

The desired outcome or ‘behaviours’ that the SLTs require are engagement within intervention sessions and effective completion of the home practice. SLTs methods of skilling up parents have been widely underreported in the literature (Klatte et al. 2020; Sugden et al. 2018). More recent studies have considered the variety of ways that are needed to support parents’ understanding of the how and the why of their children's intervention to facilitate successful collaboration (Klatte et al. 2020; Sugden et al. 2020). Our study found that to foster theirs and parents’ capabilities, SLTs work in a variety of ways, within the intervention sessions to develop parents’ skills and their own knowledge of the family. Our study highlights how SLTs draw on their own experiences, skills and knowledge, as well as life experiences, such as previous careers. Engagement with reflective practice is part of SLT's standards of proficiency (HCPC 2024) and thus a requirement of their role. Discussion with our participants highlighted how they engage with and utilise reflective practice to develop their capability with parents, drawing on skills from other areas of SLT to support work with children with SSD.

### 10.2 Opportunity

Our study found that SLTs work with parents within sessions to individualise the packages of care as recommended by the RCSLT's guidance documents (RCSLT 2018) to ensure as many parents as possible have the opportunity to benefit from intervention. Having sufficient time, resources and flexibility facilitate the SLT to do this and lack of these can be a barrier to success. Klatte et al. (2024) found that parents value SLTs taking time to collaborate with them and Sugden et al. (2019) that SLTs need to consider family contexts to allow sufficient opportunities for home practice. However, SLTs need to be cautious of mixing intervention approaches or reducing the frequency of intervention when individualising. The potential risk being that this individualisation can result in SLTs cherry picking from across the evidence, combining different techniques (Hegarty et al. 2018, 2021; Sugden et al. 2018) and potentially reducing intervention intensity based on parental preference. Further consideration is needed into the complexities between allowing flexibility and individualisation (which provides the opportunity for parents) and the need for the active ingredients of an intervention to be delivered at an appropriate intensity.

### 10.3 Motivation

SLTs perceptions and experiences of relationships with parents and their children with SSD has been relatively unexplored to date (Pritchard et al. 2024). Our study found relationships to be foundational. Relationships were found to be interlinked to all themes, which demonstrates that relationships are seen as integral to SLTs both for supporting work within therapy sessions and for facilitating effective home practice. Our findings also suggest that relationships between child‐SLT‐parent are interlinked and can be motivating to all members of the team. Previous research has found that parents also value this relationship, feeling it is important in the therapeutic process for their children with SSD and for effective home practice (McAllister et al. 2011; Sugden et al. 2019; Watts Pappas et al. 2016). When the relationship with the SLT does not work parents disengage with the intervention (McAllister et al. 2011; Watts Pappas et al. 2016), which further highlights the motivating factor of these relationships and the importance of this finding.

### 10.4 Strengths and Limitations

This paper addresses an under researched issue, bringing to light the value SLTs place on home practice for parents of children with SSD, their current clinical practice and their capability to support parents with this.

We had three clinically active SLT researchers on our team. This meant that we approached the research study bringing our own assumptions, beliefs and perspectives. K.P. who designed the study and conducted the focus groups believes that working with and supporting parents to complete home practice alongside direct intervention is a vital part of SLTs work, as well as having thoughts on what works well in this context. Whilst these views were not shared with the participants, the nature of the research project would have implied this and may have shaped the participants’ answers (or who we recruited to the study). To challenge this and bring a different perspective V.S., a linguist, acted as a critical friend to the project. Having SLTs on the research team was also a key strength. K.P. conducting the interviews allowed for specific probing due to detailed knowledge of how SLTs work in practice. It is possible that having a non‐SLT interviewer in addition would have allowed different viewpoints to be shared to strengthen the data generation further.

Having SLT participants with a variety of years of experience and who worked across different sectors and different geographical locations was another strength of our study. This comparison allowed us to consider the differences in clinical practice, the impact of different service models and challenge some of our assumptions. For example, K.P., E.P. and J.T. have worked across NHS and independent SLT services in the United Kingdom and prior to the groups held the assumption that NHS services were likely to be more restricted than independent. However, this assumption was challenged as can be seen from the results where NHS SLTs could ‘*offer children what they need*’. Being challenged in this way allowed us to learn from our participants.

One of the key limitations of our study was not probing more specifically about exactly what SLTs expected of parents in terms of home practice. We explored the importance of setting shared expectations, asked how SLTs worked with parents to support home practice and how they monitored home practice but not specifically what was expected of parents. Within these discussions some SLTs shared information about expected dose of home practice but this was not a focus of the discussions. We also did not ask how frequently they were able to provide intervention for children with SSD, although it was clear from the discussions that this varied depending on where they worked. Having this information would have allowed us to consider more critically whether SLTs were adhering to evidence‐based intervention intensity and how much of this was delegated to parents. Exploring this with SLT and parent dyads would be an interesting area for future research, allowing analysis to consider diverse approaches to working with parents, the impact of these on families and outcomes for children with SSD.

### 10.5 Conclusion

By integrating the COM‐B model of behaviour change with our findings we illustrate how this model can be used to shape SLTs clinical reasoning when working with parents, to consider how they support the development of the desired behaviours in the context of theirs and parents’ ***capability, opportunity and motivation***. We confirm the importance of relationships in supporting parental engagement, motivation and in the building of opportunities. Relationships may not only be important to parents and children but also to the motivation of SLTs who value working with parents as partners. Time needs to be taken to build collaborations effectively. Engagement of SLTs and parents within sessions supports the building of parents’ capabilities. SLTs need to draw on their skills, knowledge and experience to support them to work with parents effectively, and where these capabilities are lacking, they need to recognise that they need further support and training following graduation from university. Services that allow SLTs enough time to work flexibly and therefore individualise their provision, provide parents with the opportunity to access the service in a meaningful way. This may be important to intervention outcomes. However, caution should be exercised when considering the relationship between individualisation and adhering to the evidence.

## Policy on Using ChatGPT and Similar AI Tools

AI has **not** been used at any stage of this research or in the writing up.

## Ethics Statement

Ethical approval was gained from University of Reading Research Ethics Committee 2023‐087‐EP.

## Consent

Written and verbal consent was gained from participants following the protocol approved by the ethics committee. The consent form is available in the Supporting Information.

## Conflicts of Interest

Author Dr Jill Titterington is Editor‐in Chief of the IJLCD.

## Supporting information




**Supporting Information**: jlcd70224‐supp‐0001‐SuppMat.docx

**Supporting Information**:jlcd70224‐supp‐0002‐SuppMat.docx:

**Supporting Information**: jlcd70224‐supp‐0003‐SuppMat.docx

**Supporting Information**:jlcd70224‐supp‐0004‐SuppMat.docx

## Data Availability

Additional data is available in the supplementary materials, including the anonymised transcripts from the groups.

## Endnotes

1 Islands under the sovereignty of the British crown but outside the United Kingdom.

2 For context each quote is followed by the sector/s the SLT works in and their number of years’ experience.

3 […] indicates that some text has been removed. Text is removed when the participant added information that was not relevant to their point, such as an aside.

4 NHS is the national health service, the public health service in the United Kingdom.

5 These four participants were spread between the four groups, so each group had one member who had a previous relationship with the researchers. Participants in the groups did not know each other.

## APPENDIX 1

### FIRST AUTHOR'S REFLEXIVITY AND POSITIONALITY

All the groups were facilitated by myself, a practising SLT with 18 years' experience, a PhD student and lecturer with a specialist interest in SSD. EP was an observer in the groups, also an SLT and experienced researcher. JT is an SLT and VS a clinical linguist who are both experienced researchers with experience of qualitative research. Myself, EP and JT all have experience working with children with SSD and their parents and were aware that we have our own opinions of what works well in the context of supporting parents from our clinical experiences. I used my experience and clinical skills to facilitate the groups and to ensure appropriate follow up allowed for rich data to be gathered. Regular discussions with the whole team, including VS (who brought an ‘outsider’ perspective) was important to act as a critical friend and thus enhance my insight. The presence of experienced SLTs leading the group may have had an impact on the participants’ contributions. To ensure rigour fortnightly discussion and reflection focused on the role that our experience and our presence had on the data generation, the analysis and the final interpretation. We considered that the participants had volunteered to participate after seeing an advert and were therefore motivated to join the discussion which likely led to recruiting SLTs with a special interest in the subject. The online environment also had an impact on our data generation. Participants had to put their hand up to ensure everyone was heard and this reduced the interactive nature of groups. It was harder for participants to interject and respond in the way they may have done in a face‐to‐face setting. I kept a reflective diary throughout the process to detail this, as well as noticing my reactions to unexpected data or when views that were not in line with my beliefs.

 
[Table jlcd70224-tbl-0002]

**TABLE A1 jlcd70224-tbl-0002:** Semantic code examples.

Data extract	Code	Notes
‘Sometimes the parents need that time and that space for you, for them to then become effective, therapists at home, at home with the child’.	Successful coaching takes extra time	Here P5 is talking about needing time to succeed with a parent to support them to be more effective at home. The meaning here is very explicit and the code reflects this.
‘If we're more organised, that can help us maintain that regular contact with parents in between therapy’.	Being organised supports work with parents	Here P12 explicitly talks about organisation as being helpful to what she does.

## APPENDIX 2

### EXAMPLES OF SEMANTIC AND LATENT CODES

[Table jlcd70224-tbl-0003]

**TABLE A2 jlcd70224-tbl-0003:** Latent code examples.

Data extract	Code	Notes
‘I think I think we've been fairly successful. I think a lot of our parents have been quite engaged’.	SLTs feel confident working with parents	Here P12 doesn't explicitly say they feel confident but the fact they are speaking of success and being able to engage parents indicates a level of confidence in their ability.
‘For some of the interventions, we don't have that kind of research around the lower intensities’.	SLTs are not always aware of evidence for practice	Here P7s words imply that they are not aware of the evidence base around lower intensities that does exist.
‘We're very lucky. I think that we can give a lot’.	Being able to provide ‘enough’ direct sessions is a privilege	Here the word ‘lucky’ indicates that this level of service is not the norm and they are in a privileged position to be able to provide it. The word ‘luxury’ was also used by others in this context.
