# Supplementary material for: ‘Team Speech Sounds’—How Speech and Language Therapists Work With Parents of Young Children With Speech Sound Disorder: A Focus Group Study
Source: Int J Lang Commun Disord. 2026 Mar 23;61(2):e70224. doi: 10.1111/1460-6984.70224 (PMC13007487; doi:10.1111/1460-6984.70224)
Supplement: Supplementary file 1 — Supporting Information: jlcd70224‐supp‐0001‐SuppMat.docx [file JLCD-61-0-s001.docx]

# ‘Team Speech Sounds’ – How Speech and Language Therapists Work with Parents of Young Children with Speech Sound Disorder: A focus group study - adherence to Reflexive Thematic Analysis Reporting Guidelines (RTARG) – Braun and Clarke 2024

Adapted from original, accessed here: <file:///C:/Users/pf909356/Downloads/sj-pdf-1-pmj-10.1177_02692163241234800.pdf>

| **Advice for aspects of the research report/approach to reporting** | **Guiding notes and further explanation** | **Comments on our paper and page number/s where relevant** |
| --- | --- | --- |
| ***Background and rationale*** | | |
| Provide a robust context and rationale for the proposed research in the Introduction. | Can discuss existing research, theory, and the wider context; the researcher is understood as entering a conversation with existing scholarship. | We believe that our background section pg. 4-6 indicates where our study fits within the existing research. |
| Clearly articulate a research question – one that is methodologically coherent. | Can discuss refining an initially broader research question to a more specific one for the paper. | Detailed on pg. 6. This is methodologically coherent as we are investigating human perspectives and experiences which aligns with a qual approach and RTA. |
| ***‘Owning your perspectives’*** | | |
| Include information on guiding theoretical assumptions and other (e.g., explanatory) theory informing the use of TA | Guiding (e.g., paradigmatic, ontological and epistemological) and other theory should be coherent with RTA. | We took a subtle realism approach described on page 6 (see also info below about QR-LAW) |
| Report in a way that is consistent with stated theoretical assumptions throughout. | Theoretical coherence is evidenced through the use of language and concepts (e.g., around theme development, research subjectivity, data interpretation), the treatment of data, and use of quality practices consistent with RTA | We have used language in line with RTA e.g. ‘data generation’ ‘theme development’ ‘methodology’  We have not used positivist approaches, such as reliability checking or codebooks but have instead engaged in reflexive journaling and conversations throughout the analysis process (see pg. 8-9 and supplementary materials for examples of reflexivity) |
| Evidence methodological coherence/integrity in both the research and the report. | Theoretical assumptions, research questions, methods/practices of data generation, RTA, and specific orientation to RTA, purpose of research etc. all “fit together”, conceptually. | According to Bradbury-Jones et al (2017) **Qualitative Research Level of Alignment Wheel™ (QR-LAW)** our approach (‘generic qualitative’), focus (‘representing human experiences’), sample (purposive sample of around 12-15 participants), data collection methods (focus groups) and data analysis (thematic analysis) all align.(Bradbury-Jones et al., 2017) |
| Show evidence of reflexive practice. | Can discuss researcher professional or personal positioning and experience in relation to the topic, and/or participant group, and/or their role in shaping the research; use of reflexive journaling. | This is detailed on pg. 8 and in strengths and limitations section. For more examples of our reflexive practice see ‘description of the analysis process and reflections’ in the supplementary materials |
| Write in a methodologically coherent style. | A first-person writing style suits RTA, as it “writes in” the researcher and contributes to situated and reflexive reporting. | See throughout use of initials (taken out for blind review), ‘us’ and ‘we’ to centre ourselves in the process/decisions made. We have improved this following review. |
| ***Methodology – participants/data items*** | | |
| Describe selection of participants/data items | Should include criteria for selection and/or recruitment strategies and settings | Detailed on pg. 7 |
| Describe number of participants/data items; provide a rationale or explanation around dataset or participant group size/composition. | Non-positivist qualitative concepts, such as “information power” or sufficiency offer conceptually appropriate justifications for “dataset” or “participant group” size and composition | Detailed on pg. 7 |
| Discuss characteristics of participants/data items. | Balance the need to “situate the participant group” with participant anonymity (e.g., aggregate or report minimal demographics where appropriate). | Participant characteristics are reported in table 1. |
| Detail ethical approval and ethical code/principles followed, participant informed consent, etc. | Ethical discussion usually includes institutional ethical approval (if needed), but may include wider principles; providing research materials (participant information, consent form, etc.) in supplementary materials may be useful to support reflexive openness | Reported on pg. 6  See participant information letter, forms sent out for consent and demographic questions all included in supplementary materials |
| ***Methodology – dataset generation*** | | |
| Provide some rationale for method(s) for data generation/data item sources chosen. | Discuss why the method(s) of data generation/data source was a good fit with the research question, participant group, guiding theory, etc. If multiple data sources are used, any rationale for combination should be conceptually appropriate (e.g., crystallisation). | Detailed on pg. 6-7 - also relevant is comment in introduction on pg. 4 that existing literature is survey based and so does not have the required depth. |
| Describe development and/or characteristics of data generation tool(s). | Include tool(s) in supplementary materials when possible; discuss piloting if used, and any changes following piloting, or during data generation. | Pg 7-8  Topic guide in supplementary materials.  We did not pilot the topic guide due to the level of expertise within the team and closeness to the topic area and participants’ clinical experiences/expertise. |
| Include details such as modality and/or setting of data generation, time frame, and other pertinent procedural information. | Relevant information includes: the mode of a data generation tool (e.g., video call focus groups; chat-based interviews); the context of data generation (location; timeframe) – where this doesn’t compromise participant anonymity; and mode of recording interactive data generation. | Pg 7-8 |
| Describe who conducted any interactive data generation (which author or research role), and how. | Can include what, if anything, the researcher disclosed about their personal or professional positioning or motivation; what skills and experience they brought; note researcher’s relationship with participants prior to, during and after the research. | This is detailed on pg. 7-8 and reflected later in author’s reflexivity Pg8 and appendices.  We have indicated on the participant information table how many participants were known to the researchers who conducted the groups but have left it vague so as not to risk identifying the participants. |
| Describe the size/scope of dataset and dataset items | Such as the range and average length for interviews/focus. groups; range and average word length for textual data items. | Pg. 7-8 – details the number of and length of focus groups |
| Describe, and if relevant explain, any preparation of data for analysis. | Such as method of transcription of audio/video data (a transcription key can go in supplementary materials); changes and “corrections” – such as why typographical errors in written data were corrected; system for removing any identifying information; use of pseudonyms and/or data codes. | Pg.8 details this |
| ***Methodology - data analysis*** | | |
| Provide some rationale for use of RTA, and, where relevant, for combining RTA with other approaches and procedures. | Any combining of RTA with other method/ologies or procedures should be warranted, rather than based on a misunderstanding of RTA, and conceptually coherent (unless clearly justified). | Pg 8 details rationale |
| Describe specific orientation to RTA. | Locate RTA on dimensions of inductive<>deductive and semantic<>latent. | Pg 8-9 details this. Appendices include examples of latent and semantic codes. This is also described in more detail in the supplementary material. |
| Discuss how the researcher(s) engaged with the analytic process. | Provide a specific and situated account of the analysis process; use supplementary materials to provide a fuller account of the analytic process. | P8-9  We have provided supplementary materials and appendices to support the reader to understand our process in depth.  See ‘description of the analysis process and reflections’ in the supplementary materials |
| Where more than one person is involved, describe who analysed the data (author or research role). | Role(s) or involvement throughout the process should be discussed; where coding was collaborative, what this involved and how differences in coding and theme development were tackled, should be included. | Pg.8-9 describes the analysis process and who was involved.  See ‘description of the analysis process and reflections’ in the supplementary materials for further details. |
| Use language to describe the process and products of RTA that is coherent with the values and assumptions of RTA. | Language should convey the active role of the researcher(s) in “generating”, “crafting”, “constructing”, “creating”, “producing” or “developing” themes; language around themes should evokes them as products of a researcher-data process. | We believe our language throughout conveys the active role we had in generating the themes. We have avoided language such as ‘emerging’ ‘appearing’ etc |
| ***The analysis – reporting the data analysis*** | | |
| Provide an overview of themes or thematic structure. | Overviews can include a list, map or table of themes to preview the analysis. | See figure 1 – map of themes and subthemes for overview and links between themes and subthemes. |
| Ensure theme conceptualisation is appropriate to RTA, and any divergences are justified and explained. | In RTA, themes report shared meaning, united around a central organising concept that differs for each theme. | We have avoided theme names that are subject headings and used those which explain the central concept. We believe that each theme and subtheme are distinctive and where there is some overlap with the overarching theme it is explained. |
| Name themes appropriately. | Use theme names that capture the “essence” or “story” of each themes; brief data quotations can be used. | We have tried to avoid themes names that are merely subject headings and feel that our themes and subthemes tell the story of the data.  See ‘description of the analysis process and reflections’ in the supplementary materials for reflections on this and appendices for an example of the evolution of a theme name to best capture the essence of the theme. |
| Report themes in sufficient depth and detail. | As RTA is an interpretative method, themes should be multifaceted, and contain both data and analytic narrative; if useful, additional data extracts may be included in supplementary materials | We believe that our write up does this. Page 9 onwards. We have included some additional quotes and explanations in the supplementary material for clarity. |
| Use subtheme judiciously. | Themes are the main analytic purpose, and should be multifaceted; only use subthemes where doing so highlights an important facet or aspect of the central concept of a theme. | We have only used subthemes where we feel they highlight important nuance of main theme. We have also avoided the use of sub-headings in our write up. Since review we have revised this further to only include 2 subthemes. |
| Ensure the analytic narrative explains the meaning and significance of the data. | For RTA, each theme needs an analytic narrative that outlines its meaning and importance in relation to the topic, research question and dataset; the reader needs to be told about why/how data excerpts matter and “evidence” the theme; the Analysis section also needs to convey the overall story of the analysis. | We have combined data extracts with our own analysis to create a narrative for each theme, indicating the importance of the data to the theme and the story created. |
| Provide an appropriate balance of analytic narrative and data extracts – both data extracts and analytic narrative matter. | The rich descriptive and/or interpretative story of the analysis needs to be woven around sufficient analytic extracts from across the dataset. | We believe we have created an rich interpretative story with the themes and have included data and analysis to explain the core meaning of each theme/subtheme. We have highlighted conflicting views, even where this only came from one participant to highlight the complexity of the topic. |
| Demonstrate coherence between analytic narrative and illustrative/evidentiary data extracts. | Data extracts should convincingly and compellingly evidence the analytic claims. | We believe that our results/discussion section does this. |
| Integrate existing research and theory into the analytic narrative. | In RTA, an interpretative analytic narrative is enriched by incorporating relevant existing research and theory into the reporting of themes, reflecting notions of contextualised meaning, and contributing to an ongoing “conversation” about a topic. | Where our themes support or differ from the existing literature this is mentioned as part of the analytical narrative. Some examples can be seen on pg. 14 and 15 |
| ***The final section – quality, evaluation and conclusions*** | | |
| Draw analytic conclusions across themes. | Orient to the “so what” of the overall analysis – the “point” of the story told; this might include discussion of implications for practice and “actionable” outcomes. | In the final section we have drawn the analysis back to the research questions to indicate the point of the analysis. We have located our results within an existing theoretical framework to support the relevance of the findings and implications for SLTs |
| Discuss implications or directions for future research. | Any suggestions for future research should stem from the analysis and be evidence based (e.g., provide grounds for other groups potentially having different experiences or views) rather than generic. | We have indicated where we feel further research can be done and linked this with our analysis and current literature. For example see our discussion on pages 10-11 and 24 about the boundary between flexibility and evidence based practice. |
| Use and report quality practices coherent with RTA. | Ensure evaluation of research quality deploys conceptually coherent notions, such as: member reflections; crystallisation; others serving as a critical friend/sounding board to enhance insight; reflexive journaling. | See author reflexivity section in appendices |
| Evaluate the research from a Big Q standpoint. | Such evaluation might including considering how the specifics of the study may have shaped the research produced (for example, the characteristics and context of the participant group/dataset; the methods and modalities for generating the data); situatedness should not be treated as a limitation. | See author reflexivity section in appendices |
| Include reflections on research process and practices, including researcher reflexivity. | Some consideration of the researcher(s)’s role in shaping the research and the knowledge generated is an important quality marker. | See ‘author reflexivity’ section and ‘description of the analysis process and reflections’ in the supplementary materials for further reflections. |
