# Supplementary material for: ‘Team Speech Sounds’—How Speech and Language Therapists Work With Parents of Young Children With Speech Sound Disorder: A Focus Group Study
Source: Int J Lang Commun Disord. 2026 Mar 23;61(2):e70224. doi: 10.1111/1460-6984.70224 (PMC13007487; doi:10.1111/1460-6984.70224)
Supplement: Supplementary file 3 — Supporting Information: jlcd70224‐supp‐0003‐SuppMat.docx [file JLCD-61-0-s002.docx]

**Focus group topic guide**

This guide will be used flexibly, as per focus group methodology to build on and develop novel ideas brought to the group by the participants. This means that not all questions will be asked and the order of the sections may change to allow the questions to be answered in an organic way. The topic guide may be tweaked between each group as the ideas develop and emerge. The ‘overarching research questions’ and ‘Aim’ will remain the same and will be referred to throughout the process to ensure that each focus group has a clear focus.

# The ways in which speech and language therapists work with parents of children up to age 5;11 with speech sound disorders (SSD).

**Aim:**

To explore the current practices and perspectives of practising SLTs working with children, up to age 5;11, with SSD and their parents. Focusing on how SLTs form effective working relationships with parents and the techniques and strategies they use to support parents to carry out effective intervention at home with their child.

**Overarching research questions:**

1. What are the perceptions and experiences of SLTs about the way they currently work with parents, within therapy sessions, to ensure that the approach and intensity of intervention are delivered with fidelity in the home environment?
2. What do SLTs think works well to build relationships with parents to support them with home practice for SSD therapy?
3. What do SLTs think are the barriers and facilitators to supporting parents effectively?

**Introduction:**

Prior to group, participants will have been sent an information sheet, consent form and demographic information. They will have a chance to ask any clarifying questions by email or phone. The information gathered will be used to purposively sample diverse groups to allow for a range of opinions and experiences.

Thank everyone for taking part, remind participants of the purpose of the group, aims of the research and how results will be used, introduce self and 2^nd^ investigator. Confirm that participants understand what is involved, provide opportunity for questions and gain verbal consent that they are happy for the meeting to be recorded. Start the recording and reconfirm for the recording that everyone is happy to be recorded.

Go through the code of conduct for the group:

| **Code of conduct**  The focus group is a safe space where we hope you will feel comfortable sharing your views. We ask that everyone follows the code of conduct below:   - We understand that people may have different views. Please respect the views of others even if you disagree with them. - Please do not use inappropriate or disrespectful language. - Please do not share personal information disclosed by others outside of this session. - Please keep your microphone on mute until it is your turn to speak to reduce background noise. - If you’d like to speak, please raise your hand on Teams. - Please do not send private messages or comment in the chat to ensure we are all aware of all the conversations that are happening. - If you need help for any reason you may private message the member of the team who is providing tech support (specify who this is for participants) - Please do not forward on any invitations to this session. Only those who have received a confirmation will be allowed in the session. - Please have your camera on, body language and facial expressions may be important for interpreting what has been said. If this is not possible due to internet connection then please do let us know. - You do not have to answer any questions you do not want to. - You are free to leave the session at any time, or to take a break.   If someone is not following the code of conduct, we will remove them from the session and follow-up with them after. |
| --- |

Make sure everyone is clear that we are talking about working with parents of children with SSD aged up to 5;11.

Allow time for participants to introduce themselves to the group (name, where they work, thoughts/experience of working with SSD).

**Ice breaker question** – The aim of this is to get the group comfortable talking to each other on an everyday topic and will be a chance to practice using the hands up, reactions and microphone functions. Something along the lines of…What is your favourite food? Where is the last place you went for the first time? Last thing you watched on TV?

**Opener**

This section aims to start the group talking about the specific topic, initially in quite a general way before getting into the specific research questions.

- ***What are your experiences of working with parents of children with SSD when delivering intervention?***

How much does your experience influence what you do? How comfortable/confident are you working with parents?

** KP to summarise and conclude what the therapists have said and confirm understanding of information.*

**Section 1 (aims to address question 1)**

- ***How do you work with parents in therapy sessions to support practice at home?***

Possible probes – what factors do you think are important? How do you set expectations with parents about home practice? do you provide anything in writing? How do you check in on parent’s understanding? Do you ask parents to have a go at therapy in the session?

Show participants quotes to spark discussion on the topic

***‘Having parents carry out speech-language pathology tasks at home was identified as a possible way of increasing intervention intensity’ -*** Hegarty et al 2021, p. 219

***‘Involving parents in therapy offers a potential solution to the lack of clinical time … A concern here is that too great a reliance on parents might disadvantage those children whose parents are unwilling or unable to participate.’*** – Joffe and Pring 2008, p. 160

- ***How do you monitor/ensure that a parent is completing the homework as you have recommended?***

Possible probes – if you don’t feel a parent is completing homework what can you do/do you do? How do you support those parents that find the therapy harder to access/engage with? How can we make our intervention effective and accessible for all?

** KP to summarise and conclude what the therapists have said and confirm understanding of information.*

**Section 2 (aims to address question 2)**

- ***Which of the following best describes how you set up your sessions with parents with children with SSD?***

1. Parent/s sits at the side or edge of the room
2. Parent/s sit at the table or on the floor with me and the child
3. Parent sits at the table with the child and I sit back
4. Parent/s don’t come into the room
5. Other

Possible probes –What are your thoughts about how important working with the parents is?

- ***How do you think your relationship with parents impacts on how you support them with home practice?***

Possible probes – does it improve outcomes – why/why not? is parental ‘buy-in’ needed for successful therapy? Is a good relationship needed with a parent?

- ***How do you work towards building a successful relationship with parents?***

Possible probes – is there any way to develop a positive relationship after a negative first experience? How important is the model of service delivery/continuity of therapist in parental engagement?

- ***How do you know when a parent is engaged with therapy?***

Possible probes - What training have you received to support your work with parents? What training would be/would have been useful to you?

** KP to summarise and conclude what the therapists have said and confirm understanding of information.*

**Section 3 (aims to address question 3)**

Show participants quotes to spark discussion on the topic

**‘it’s not so much that’s what she’s paid for… but she’s the speech pathologist, so she’s to do the work.’** - Watts-Pappas et al 2016, p.231

***‘there was very little input from me, she just sort of took it and asked him to say words and played games but didn’t ask me much at all.’*** - Watts-Pappas et al 2016, p. 233

- **What facilitates you to work well with parents?**
- **What stops you from working effectively with parents?**

Give therapists a chance to come up with own suggestions and then follow up with a summary of the barriers discussed in the literature and see what SLTs think.

e.g. Watts-Pappas 2018

Barriers to involving parents as reported by SLTs:

- - Workplace (e.g. service restrictions)
  - SLT (e.g. limited time, experience, confidence)
  - Parent (e.g. capability, time, attitude, confidence)

AND

Sugden et al 2018

SLTs reported influencing factors such as…

- - Service based (waiting lists, policy, size of caseload…)
  - Clinician based (experience, what always done, knowledge of specific therapy...)
  - Client (family preference, severity of disorder, age…)

** KP to summarise and conclude what the therapists have said and confirm understanding of information.*

**Summary**

- **Any final thoughts or comments before we close?**

Thank participants for coming, let them know about the next phases of the project and how this will contribute. Ask for anyone who would be interested in co-creation workshops to let me know if they haven’t indicated this on the form.
