# Supplementary material for: ‘Team Speech Sounds’—How Speech and Language Therapists Work With Parents of Young Children With Speech Sound Disorder: A Focus Group Study
Source: Int J Lang Commun Disord. 2026 Mar 23;61(2):e70224. doi: 10.1111/1460-6984.70224 (PMC13007487; doi:10.1111/1460-6984.70224)
Supplement: Supplementary file 4 — Supporting Information:jlcd70224‐supp‐0004‐SuppMat.docx [file JLCD-61-0-s003.docx]

# Description of analysis process - what we did

A six-phase reflexive thematic analysis was used to guide our analysis process (Braun & Clarke, 2021). As recommended, this was used as a guide with the analysis moving backwards and forwards between the stages as thinking developed throughout the process. All of the analysis was performed by XX [removed for review]. A reflexive diary was kept throughout the process and regular discussion with the rest of the research team supported the process. Throughout the process I found it really helpful to change locations and the medium with which I was working, taking time to go away and then come back to it a few days later. This document describes how we went about using this process and what actually happened at each stage:

## Phase 1 – familiarising myself with the dataset

Whilst there was some initial activity specifically to familiarise myself with the dataset as detailed below this ‘phase’ ran throughout the whole analytical process. Throughout the process I read and re-read the data many times over and checked my analysis back to the data at each point. This allowed me to become intimate with the data and ensured the analysis was true to my interpretation of the data itself.

#### Initial familiarisation of the data occurred through:

- Running the groups
- Checking through the automatic transcription, making corrections and anonymising the data (taking out names participants had used, locations they had spoken about and giving each participant a p#)
- Re-watching the groups to look for body language, tone, facial expression and any other non-verbal cues participants may have used that supported the interpretation of the words.
- Reading the transcripts through and making notes in the reflexive diary.

#### During these this phase I noted/reflected on the following:

- I recognised that I had different personal reactions to different groups. For example, there was one group where the participants seemed to write off the clients who couldn’t access their service. I noted that this made me feel cross. I spoke about this with the team and reflected that these views were different to my own.
- There were conflicting views from participants both within groups and across different groups. I reflected on my potential influence over people’s answers. I considered power balance – I am not anyone’s manager but there may be a sense from the participants that my role as a researcher means I have more knowledge of the evidence base, and this may have influenced the answers.
- At no point did I tell participants of my thoughts or beliefs on the subject but I reflected that the fact that I am doing research in this area and plan to create a package to support SLTs with their work with parents indicates to them that I believe strongly that working with parents is the right thing. This may have led to participants feeling like they could only discuss their best practice in this area and it my not have uncovered how they work all the time. My questions are looking for best practice though (e.g. what works well) and so this in itself may support the research questions to be answered best.
- Participants that came forward for the study mostly had a special interest in SSD, this likely self-selection of participants may have led to me attracting those who are expert in working with this client group which does not necessarily reflect the whole of the SLT workforce who work with these children. I reflected on the potential benefits and disadvantages of this to the study. For example the study will be informed by experts in the field, however the motivations of other SLTs in the field may be very different and the analysis may not speak to them/feel clinically useful to them.

## Phase 2 – coding

I started this phase using Nvivo, using the software to assign codes to the data. In some ways this process was useful and allowed me to see data extracts in organised groups, supporting my thinking. However as I moved through the process and generated more codes I found this to be too far removed from the original transcripts and so I abandoned Nvivo and started coding again using comments on MS word, alongside a table of codes and extracts. Whilst this was more time consuming in some ways it allowed me to stay true to the data and supported the familiarisation process.

Analysis at this stage was inductive to allow novel ideas to come through the data without any preconceived ideas about what I would find. I recognised that due to my familiarity with the existing literature this was not easy and so conversations with the team were to check in that my analysis was coming from the data, not my pre-conceived ideas. I was open to developing both latent and semantic codes as the research questions lend themselves both to learning from what SLTs do as well as how they feel about it. I recognised that feelings are not always explicitly said but are inferred and this therefore lent itself to latent coding. I went back to the codes throughout the remaining phases and was open to codes changing as I became more familiar with the data and clearer on my analytical take. At the end of this stage I noted what I had observed to be some key words to come out of this phase of analysis.

Table 1 - Semantic code examples:

| **Data extract** | **code** | **notes** |
| --- | --- | --- |
| ‘sometimes the parents need that time and that space for you, for them to then become effective, therapists at home, at home with the child’ | Successful coaching takes extra time | Here P5 is talking about needing time to succeed with a parent to support them to be more effective at home. The meaning here is very explicit and the code reflects this. |
| ‘if we’re more organised, that can help us maintain that regular contact with parents in between therapy.’ | Being organised supports work with parents | Here P12 explicitly talks about organisation as being helpful to what she does. |

#### Table 2 - Latent code examples:

| **Data extract** | **code** | **notes** |
| --- | --- | --- |
| ‘I think I think we’ve been fairly successful. I think a lot of our parents have been quite engaged.’ | SLTs feel confident working with parents | Here P12 doesn’t explicitly say they feel confident but the fact they are speaking of success and being able to engage parents indicates a level of confidence in their ability. |
| ‘for some of the interventions, we don’t have that kind of research around the lower intensities.’ | SLTs are not always aware of evidence for practice | Here P7s words imply that they are not aware of the evidence base around lower intensities that does exist. |
| ‘We’re very lucky. I think that we can give a lot.’ | Being able to provide ‘enough’ direct sessions is a privilege | Here the word ‘lucky’ indicates that this level of service is not the norm and they are in a privileged position to be able to provide it. The word ‘luxury’ was also used by others in this context. |

#### During this phase I noted/reflected on/felt the following:

- I observed that despite my inductive coding I noted some parallels with exiting literature. I used team meetings to discuss whether these were really in this data or if I was allowing my prior knowledge to influence my coding.
- I reflected on how my beliefs were influencing my codes. For example when SLTs spoke about being sent on training to support their work with parents I interpreted this to mean that services value and see the importance of working with parents. Another interpretation could be that services see working with parents as a way to reduce direct contacts and thus save money.
- Participants had experience working for a variety of services. My own experiences are working for the NHS and university clinic. I have no experience of providing private SLT, I reflected on any potential internal bias’s that I may have and considered whether this impacted my interpretation.

## Phase 3 – generating initial/candidate themes

I started this phase manually by printing out and cutting out all the codes and laying them on the floor in my lounge. I organised them into groups with shared meaning. This manual process allowed me to move codes between groups whilst I considered what the shared meaning of the groups were. I found it easier to do it this way as I had an overview of everything at once. Once I was happy with the organisation of the codes I wrote a summary of the key points under each theme to support me to bring my initial thoughts to the rest of the team. This process led to me moving some of the codes around. The team asked me questions about my initial themes and this challenged me to really think about how the codes fit the themes. Again following discussion I moved some of the codes around, as well as renaming some to fit my thinking better. At this point I started thinking about my research questions and how the themes might relate to answering these.

#### During this phase I noted/reflected on/felt the following:

- I found this very overwhelming initially, there seemed like a lot of codes and on the first viewing it seemed that there were a lot that I couldn’t imagine fitting into themes. I felt frustrated that I had 3 research questions as this made it seem even harder. It helped to just get started, at first inductively without consideration of questions, making the initial groups and then moving things between them. From here I considered the research questions and this felt more manageable.
- I found it very easy to get distracted thinking about how some of the more practical elements could relate to the next phase of my PhD.

## Phase 4 and 5 – developing and reviewing themes. Refining, defining and naming themes

During phase 4 I went back to the data. I created a table that was arranged by each theme/sub-theme with all the codes and the corresponding data. Again, at this phase there was movement between the codes and themes to ensure that my analysis was viable. After I had completed the table the rest of the team reviewed it and asked questions and made comments. This was invaluable to make sure that I was happy with my interpretation. Being able to talk it through helped to develop my thoughts and deepen the analysis. I then tried again to write a summary. At this phase some of the themes changed names to better reflect the analysis and better explain the story behind the theme. I found that phase 4 & 5 merged into each other and one very much influenced the other and the fine tuning in phase 5 was very much influenced by the development in phase 4. During phase 5 I developed names that I felt happier with in terms of their ability to tell the story of the analysis.

#### During these phases I noted/reflected on/felt the following:

- Some themes seemed close to previous studies with SLTs, but others were novel findings. This may be due to nature of previous studies with SLT which have all been survey based. There is very little existing research, so this lends itself to a more inductive analysis.
- I felt excited at this phase as I started to really make links to the next phase of my research and feel the value of completing this study.
- By phase 5 I could clearly see how the themes related to my research question, as this became clearer, I felt happier with my analysis.
- I was still not happy with the name of theme 2 but decided that I should start on the write up in the hope that the writing process made the theme clearer. See figure 1 for evolution of theme 2.

#### Figure 1 - Evolution of theme 2 (also appendix 2 in main document)


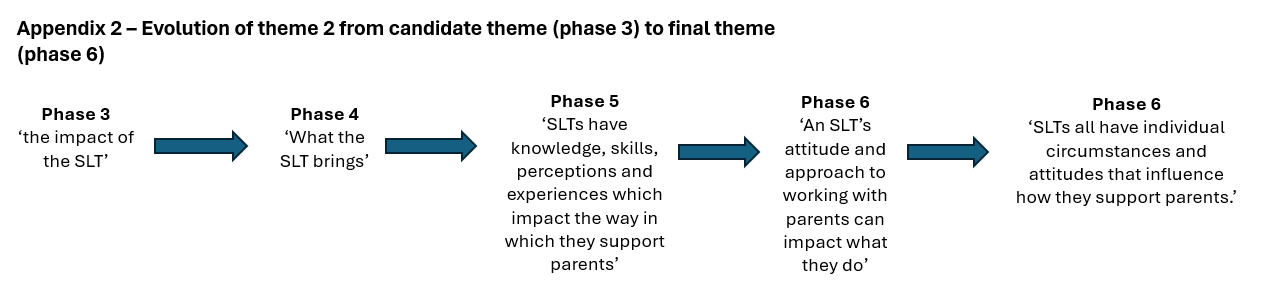


## Phase 6 – writing up

During this phase I continued to develop and deepen the analysis, including and then building a narrative around the data extracts in a way that adhered to word counts and provided clarity for the reader was extremely difficult and I hugely benefitted from discussion, feedback and suggested edits from the whole team. Throughout this phase the discussion continued and some theme names were tweaked as a result of the discussion and writing up process. Towards the end of the phase I used RTARG tool(Braun & Clarke, 2024) (see supplementary materials),

#### During this phase I noted/reflected on/felt the following:

- At times I found the process incredibly frustrating, I would get to a stage I was happy and then others in the team had suggestions or it wasn’t clear to them. Ultimately this was very helpful
- Towards the end of write up I found it particularly difficult and attempted to start the write up from scratch but this just resulted in the word count getting too big. It took many drafts until we were happy with the end product.
- Throughout the write up I kept thinking back to the wonderful participants and how they took their time to share their experiences and knowledge, I felt really grateful and want to do them justice and get their voices heard. I found it really hard removing quotes as I synthesised the write up, knowing that I had to miss out some excellent quotes as there are just too many to include.
- The RTARG was helpful to check what I had written was in line but I found the suggestions made it very hard to keep to the word count. Something I need to practice!

## References

Braun, V., & Clarke, V. (2021). *Thematic Analysis: A Practical Guide* (1st ed.). SAGE.

Braun, V., & Clarke, V. (2024). Supporting best practice in reflexive thematic analysis reporting in *Palliative Medicine* : A review of published research and introduction to the *Reflexive Thematic Analysis Reporting Guidelines* (RTARG). *Palliative Medicine*, *38*(6), 608–616. https://doi.org/10.1177/02692163241234800
